# Using ANN for thermal neutron shield designing for BNCT treatment room

**DOI:** 10.1038/s41598-024-65207-w

**Published:** 2024-06-26

**Authors:** Fatemeh S. Rasouli, Atefeh Yahyaee, S. Farhad Masoudi

**Affiliations:** Department of Physics, K.N. Toosi University of Technology, P.O. Box 15875-4416, Tehran, Iran

**Keywords:** BNCT, Artificial neural network (ANN), Composite shield, Treatment room, Monte Carlo simulation, Applied physics, Biological physics

## Abstract

Occupational radiation protection should be applied to the design of treatment rooms for various radiation therapy techniques, including BNCT, where escaping particles from the beam port of the beam shaping assembly (BSA) may reach the walls or penetrate through the entrance door. The focus of the present study is to design an alternative shielding material, other than the conventional material of lead, that can be considered as the material used in the door and be able to effectively absorb the BSA neutrons which have slowed down to the thermal energy range of $< 1$ eV after passing through the walls and the maze of the room. To this aim, a thermal neutron shield, composed of polymer composite and polyethylene, has been simulated using the Geant4 Monte Carlo code. The neutron flux and dose values were predicted using an artificial neural network (ANN), eliminating the need for time-consuming Monte Carlo simulations in all possible suggestions. Additionally, this technique enables simultaneous optimization of the parameters involved, which is more effective than the traditional sequential and separate optimization process. The results indicated that the optimized shielding material, chosen through ANN calculations that determined the appropriate thickness and weight percent of its compositions, can decrease the dose behind the door to lower than the allowable limit for occupational exposure. The stability of ANN was tested by considering uncertainties with the Gaussian distributions of random numbers to the testing data. The results are promising as they indicate that ANNs could be used as a reliable tool for accurately predicting the dosimetric results, providing a drastically powerful alternative approach to the time-consuming Monte Carlo simulations.

## Introduction

Boron neutron capture therapy (BNCT) is a promising two-step cancer treatment method that involves delivering $^{10}$B in the tumor and irradiation of neutrons with appropriate energy toward the target which induces a nuclear reaction that releases high-LET particles, i.e. the particles with high linear energy transfer, of alpha and $^7$Li nuclei^1^. These particles deposit, on average, 2.34 MeV of energy within a 10 $\mu$m range and destroy the tumor cells which are labeled by boron nuclei. This targeted approach leads to the selective destruction of tumor cells and reducing the risk of side effects to surrounding healthy tissue. BNCT has been of interest to scientists, clinical doctors, and patients since early 1950 and has been trying to fulfill the dreams of treating tumors^2^. A complete discussion on this treatment method is beyond the scope of the present work and there are worthy studies available on various aspects of BNCT including beam designing^3–10^, dose calculations^11–14^, medical aspects^15–17^, etc.

As a worthy example of the related projects in the world, one can refer to BNCT project in Studsvik facility based on the R2-0 reactor to produce a large uniform field of neutrons to reach a clinical beam for radiobiological purposes. There are many of published works addressed and solved problems related to BNCT treatment within this project^18–20^. Also, there are documents dealing with the design and optimization of the BNCT treatment rooms considering appropriate radiation shielding owing to the fact that protecting the facility personnel and the general public from radiation exposure is a primary safety concern in radiation therapies. The shields may be designed for choosing appropriate materials and their dimensions and geometries, the monitoring window for the patient position, the maze, and the entrance door of the room^21,24–27^.

In BNCT of deep-seated tumors, the patient is irradiated with epithermal neutrons (1 eV $<E<$ 10 keV) at a rate of about $10^9$ n.cm$^{-2}$.s$^{-1}$. These neutrons are then thermalized in the tissue and captured by boron (mostly accumulated in tumor) through the $^{10}$B(n,$\alpha$)$^7$Li reaction^28^. Therefore, the initial beam exited from the beam shaping assembly (BSA) belongs to the epithermal range, and is expected to slow down to the thermal energy range ($E<$ 1 eV) after passing through the walls and the maze toward the door. Consequently, designing a thermal neutron shield for the door of the BNCT treatment room is of high importance. However, estimating dose distributions outside the BNCT treatment room is a complex task due to the presence of low-energy neutrons coupled with photons in the spectrum near the entrance door. This is because thermal neutrons are absorbed into the nucleus, which leads to the excitation of the nucleus followed by the emission of $\gamma$-rays. Therefore, Monte Carlo simulations provide a suitable perspective for selecting the optimal shield by simulating the interactions of particles with matter in a detailed and accurate manner.

The materials enriched with hydrogen and other low-Z elements can cause effective energy loss of neutrons during elastic scattering. Concrete is often used as an effective and affordable material for radiation shielding in facilities that use radiation-generating equipment, such as treatment rooms. Furthermore, the slow neutrons are absorbed using neutron-absorbing elements with relatively large neutron absorption cross sections. Therefore, for effective shielding of thermal neutrons, which in the case of the entrance door of the treatment room is needed, it should be composed of elements with a large absorption cross-section for low-energy neutrons^21–23^. In this way, boron compounds have been proposed so far to be used as thermal neutron shields^29,30^.

The aim of the present work is to design and optimize a thermal neutron shield for the entrance door of a typical BNCT treatment room based on the shielding material proposed by Shahram et al.^31^. The Geant4 Monte Carlo code has been used for designing the geometries and transport of the particles in the medium. Owing that the optimization is a complicated process involving both optimization of the shield material and its thickness/geometry, it can be managed in successive steps in the traditional Monte Carlo simulations which not only does not allow simultaneous optimization, but is also time-consuming and computationally expensive. To overcome these limitations, an artificial neural network (ANN) was employed to find an optimal mixture of elements in the composite in the shield that improves thermal neutron absorption and reduces the dose behind the door. By providing appropriate input data to the machine, the ANN serves as a powerful tool for predicting the behavior of desired parameters/quantities, allowing researchers to avoid performing Monte Carlo simulations for all possible cases. This is because of the ANN’s ability to construct a complex nonlinear mapping through a set of input and output data making it able to suggest the outcomes from the provided information of simulations/experiments. It is obvious that validating the generated results is required.

The outline of the paper is as follows: Using a previously designed neutron beam and simulation of a typical BNCT treatment room based on an existing room in IKC Hospital in Tehran, introducing the proposed shielding material, investigating the dose behind the entrance door, performing Monte Carlo simulations to generate an appropriate set of data, employing ANN to find a model to predict the dose and the desired quantities, and choosing the optimized shield considering the recommendations limits for occupational radiation protection.

## Methods

### Monte Carlo and ANN

Monte Carlo is known as a mathematical technique that employs statistical sampling for numerical experiments using the computer to estimate outcomes for uncertain events in which the deterministic methods can not give reliable predictions. This method works based on random sampling and is the best way known to explore the behavior of complex systems and geometries with multiple degrees of freedom, such as the transport of the particles in a medium. By repeatedly performing Monte Carlo simulations, many probable outcomes will generate, which become more accurate as the number of inputs grows. Finally, this method offers a clear picture including the results and the corresponding uncertainties. The necessity of performing a complete and trustworthy Monte Carlo computational model is to be used for planning the experimental work and studying possible additional optimization and improvements of the facility. Despite all these advantages, Monte Carlo is time-consuming as there is a need to generate a large number of sampling to generate reliable output, particularly for large and multi-dimensional problems. This is also the case with our problem of optimizing the thermal neutron shield for the entrance door of the treatment room. This is because the problem involves a large volume of matter through which particles must travel, leading to an increased probability of particle loss and complex interactions between particles and the medium. In addition, the problem requires the optimization of multiple parameters, such as the material composition and thickness of the shield, which are coupled and can influence each other’s effectiveness.

In recent years, the ANN tool has found widespread applications in nuclear engineering to predict the behavior of the system by assigning a model to the input data. This involves training the network using a large amount of data to learn the patterns and find relationships between the input data and the corresponding output. Once trained, the ANN can be used to make predictions or classify new data based on its learned knowledge. Considering the advantage of using ANN for predicting the results on one hand and the disadvantage of performing Monte Carlo simulations on the other hand, which involves the difficult and time-consuming task of testing all possible mixtures and thicknesses to find the optimal one, using the data generated by the Monte Carlo simulations as inputs of the ANN was proposed. It is worth mentioning that ANN is a method of predicting the results from the input data in the case of multiparameter, complicated problems having certain efficiency advantages, and is inherently different from the Monte Carlo method that uses a broad class of computational algorithms to obtain numerical results. Feeding the ANN with the Monte Carlo data and validating its generated results with proper Monte Carlo calculations must be finally done.

### Simulations

**Figure 1:**
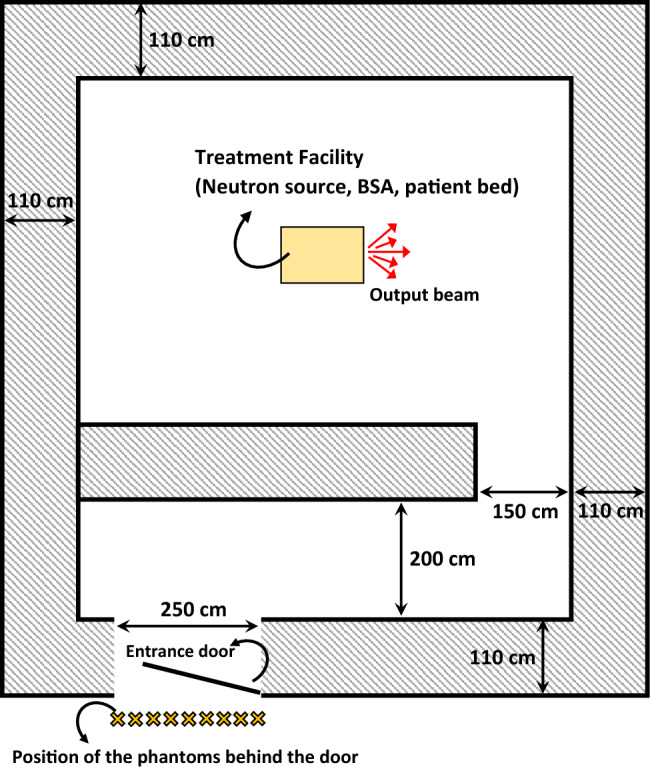
The schematic top view of the simulated BNCT treatment room. The room dimensions, the thicknesses of the walls, the output beam direction, and the position of the water phantoms for dose evaluation have been shown. The phantoms are numbered sequentially from left to right by 1 to 9.

In this work, the Geant4 Monte Carlo code was employed to perform the simulations. This toolkit is a general-purpose Monte Carlo radiation transport code that is capable of tracking various particle types including leptons, photons, hadrons and ions in arbitrary three-dimensional configurations of materials and geometries over wide ranges of energies. Also, the important features that make this code interesting include being easy to use, flexible structures, and an extensive collection of cross-section data. Moreover, Geant4 provides visualization drivers and interfaces, graphical user interfaces, and a flexible framework for persistency^32^. The other interesting aspect of GEANT4 is that it has grown over the years and changes have been made to accommodate the needs of the users so that can cover a large number of experiments and projects in a variety of application areas. Details can be found in the literature^33^. The equipment chosen for simulation was the BSA designed for BNCT based on the D-T neutron generator yielding $5 \times 10^{12}$ neutrons per second, with apertures of 6 cm radius for emission of the neutron spectrum. This equipment was simulated in accordance with the BSA proposed in our previous work^34^ for the treatment of deep tumors with the output flux of $\sim 10^9$ n.cm$^{-2}$s$^{-1}$. This system was placed in the center of the simulated treatment room based on an existing room in Imam Khomeini Hospital Complex in Tehran. The simulated treatment room featured in Fig. 1 had a square geometry of 11 $\times$ 11 m$^2$, with a maze and entrance door, and a height of 2.5 m. The concrete was considered as the material of both the main walls (those surrounding the BSA exit and the patient bed) and the secondary walls (those behind the maze). The entrance door, with dimensions of 1.5 $\times$ 2 m$^2$, was initially assumed to be made of lead in the primary simulations. The thicknesses of the walls, the beam direction, and the position of the phantoms for dose evaluation have been depicted in Fig. 1.

To assess the effectiveness of the designed shield in limiting radiation exposure, the maximum permissible doses based on the widely accepted recommendations were considered. For this purpose, nine spherical simulated water phantoms with a radius of 15 cm were placed behind the entrance door, as the present study focused on the shielding design for this area. The International Commission on Radiological Protection (ICRP) and National Council on Radiation Protection and Measurements (NCRP) publish recommendations for occupational dose limits. The NCRP limits generally agree with ICRP recommendations for dose limits and there are two types of occupational dose limits in these guidelines, including limits for specific organs or tissues and acceptable risk levels for cancer induction^35,36^. According to these standards, the weekly limits of effective dose in controlled and uncontrolled areas are 0.1 mSv/week and 0.02 mSv/week, respectively. Taking these in to account, the shielding design was planned to ensure that the maximum allowable dose rate behind it (as an uncontrolled area) is less than 0.5 $\upmu$Sv h$^{-1}$, assuming that the clinic plans to operate for 40 h per week.

The neutron and photon doses have been calculated by scoring the ambient dose equivalent in the simulated phantoms, defined as a weighted radiation dose that takes the quality factor of the particles depositing energy in biological matter into account. To this aim, whenever a neutron or a gamma ray traverses the phantom, the fluence spectrum inside the sphere is obtained and the fluence conversion coefficients are applied. All simulations tracked 5 $\times$ 10$^8$ histories, and the statistical errors associated with the results were reported.

### Shielding material

The thermal neutron shield proposed in this study has been inspired by the work of Shahram et al.^31^, who experimentally designed a polymer composite based on PMMA (polymethyl methacrylate with chemical formula of C$_5$H$_8$O$_2$ and density of 1.1 g cm$^{-3}$) and polyethylene powder (with chemical formula of C$_2$H$_4$ and density of 0.9 g cm$^{-3}$). In these materials, hydrogen capturing of thermal neutrons is through the $^1$H(n, $\gamma$)$^2$H reaction with a cross section of 0.33 barn^37^. An in-situ polymerization technique was employed to increase the composite’s slowing-down feature and the boric acid powder (with chemical formula of BH$_3$O$_3$ and density of 1.44 g cm$^{-3}$) was added to absorb thermal neutrons through $^{10}$B(n, $\alpha$)$^7$Li reaction. The produced heavy particles can easily stop in the shielding material and, therefore, have not been considered in the dose calculations. In their work, a polyethylene layer was used as a moderator, followed by a polymer composite layer as an absorber. In the second layer, boron was added at weight fractions of 1%, 5%, 7%, and 10%. In order to evaluate the effectiveness of the designed shield, various combinations of thicknesses for the two layers and the proportion of boron in the polymer composite were experimentally tested. For these limited samples, the neutron doses were measured.

Though their study was pioneering in designing thermal neutron shields, the limited number of models tested raises the question of whether there exist other combinations, thicknesses, or weight fractions that could result in even better shielding properties. To address this question and take advantage of the benefits of ANN as discussed earlier, we tested 720 different models in the present study. It is necessary because, as the number of inputs increases, the accuracy of forecasts by ANN tends to improve, making it necessary to consider a larger number of inputs. The weight fraction of polyethylene in the polymer composite, the weight fraction of boric acid in the polymer composite, the thickness of the first layer of the shield (polyethylene), and the thickness of the second layer (polymer composite) were considered as the parameters with the specific values presented in Table 1. From here, these parameters are labeled by A, B, C, and D, respectively. By using these values, 720 sets of arrangements were generated. In our simulations, each shield has been placed in front of a typical neutron source which has a neutron energy range from a few eV up to 10 MeV, and the dose and flux beyond the shield were calculated.

**Table 1:** The values assigned to the four chosen parameters to generate various sets of arrangements.

Weight fraction of polyethylene (%)	5	10	20	35	-	-
Weight fraction of boric acid (%)	10	20	30	40	50	-
Polyethylene thickness (cm)	2	4	6	8	10	12
Polymer composite thickness (cm)	1	2	3	4	5	6

### Artificial neural network

We utilized ANN for predicting both the thermal neutron flux behind the designed shield as well as the dose calculations. For each network, we had a total of 720 data sets, which have been divided into two parts: training data (690 samples) and testing data (30 samples) to validate the results. Table 2 lists the combination of the four parameters of A to C for the 30 samples that have been used as the testing data. These sets of parameters are chosen so that, in a good approximation, incorporate all the values in the ranges presented in Table 1.

**Figure 2:**
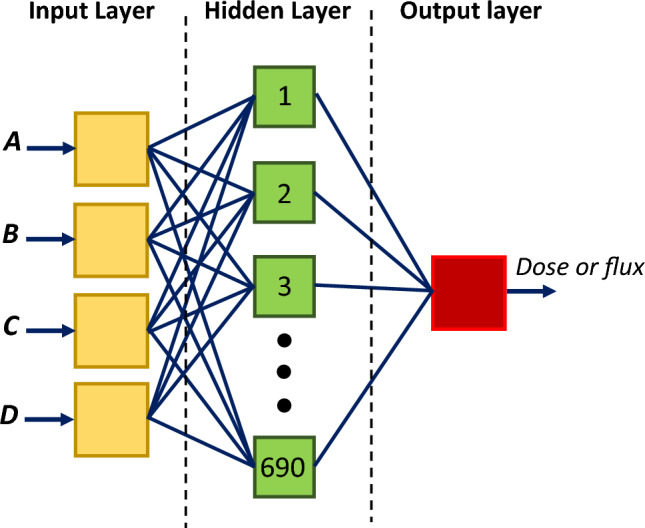
The proposed ANN architecture.

The neural network used in this study for thermal neutron flux consists of a single hidden layer perceptron with 40 neurons and an output layer. The multilayer perceptron (MLP) network has been trained using the Levenberg-Marquardt (LM) algorithm which is an appropriate option for solving generic curve-fitting problems. To calculate the output of a node based on its set of specific individual inputs and their weights, the activation function is needed. In this work, the sigmoid activation function was used for each layer. The input data for the network was a $4 \times 690$ matrix which included the neutron flux extracted from the code, and the output data was in the form of a $1 \times 690$ matrix. For the dosimetric data (a $4 \times 690$ input matrix), a two-layer Feedforward Backpropagation neural network with a hidden layer of 30 neurons was used, and trained using the LM algorithm. Also, the sigmoid activation function was used for each layer of the network. During the training phase of both networks, the neural network was initially trained with training data, and some parameters such as weights and biases have been regularized to prevent overfitting that occurs if the model cannot generalize and fits too closely to the training dataset. The networks have been trained for 1000 epochs to ensure that they had converged to a stable solution. Figure 2 shows the architecture of the neural network used in this work.

**Table 2:** Sets of the four parameters of A (the weight fraction of polyethylene), B (the weight fraction of boric acid), C (the thickness of the polyethylene), and D (the thickness of the composite) as 30 samples that have been used for testing data.

Sample number	A (%)	B (%)	C (cm)	D (cm)
1	5	10	8	1
2	5	20	2	3
3	5	20	6	4
4	5	30	2	1
5	5	30	6	3
6	5	40	8	3
7	5	40	10	4
8	10	10	2	1
9	10	10	10	1
10	10	20	6	4
11	10	30	8	3
12	10	30	10	4
13	10	40	10	2
14	10	50	4	2
15	20	10	2	4
16	20	10	8	3
17	20	20	6	1
18	20	20	10	2
19	20	30	6	2
20	20	40	6	1
21	20	40	8	1
22	20	50	6	2
23	35	10	2	2
24	35	10	10	4
25	35	20	6	2
26	35	20	10	3
27	35	30	10	2
28	35	40	4	2
29	35	50	4	4
30	35	50	6	3

## Results and discussion

**Figure 3:**
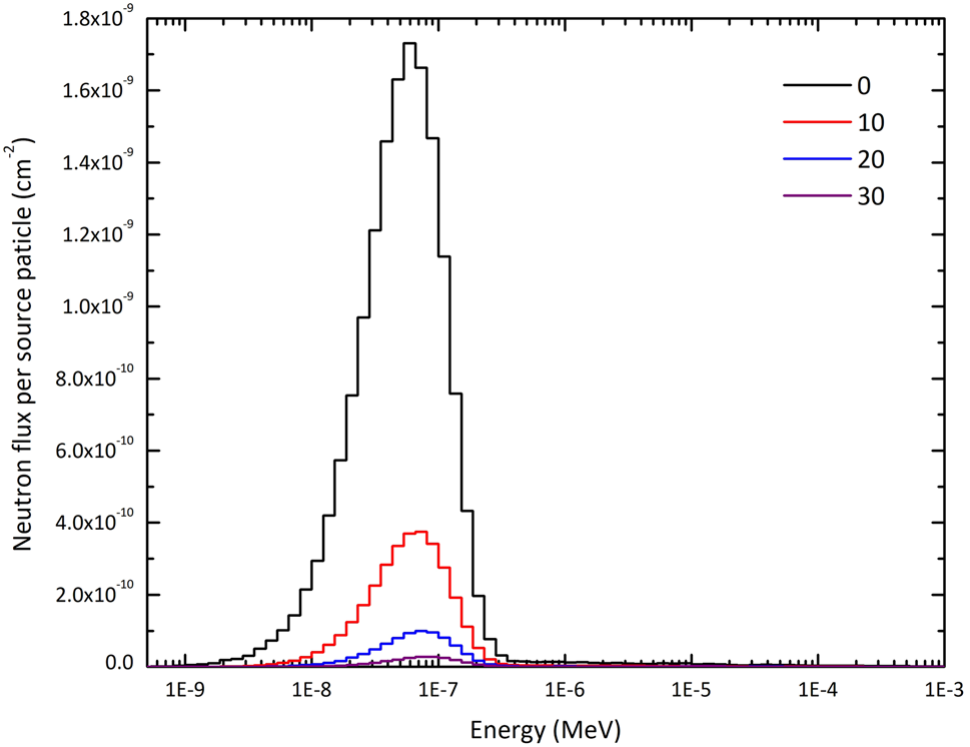
The neutron spectrum behind the entrance lead door of three different thicknesses for the treatment room (see Fig. 1). The thickness of 0 cm refers to the absence or being open of the door. The initial neutron spectrum exiting from the BSA has been taken from Ref.^34^. The neutron fluxes have been reported per neutron source particle.

Figure 3 displays the neutron spectrum per neutron source particle behind the entrance door of lead with three different thicknesses, and a comparison was made with the neutron spectrum corresponding to the absence or being open of the door. The results indicate that despite the initial spectrum exiting from the BSA beam port being in the epithermal energy range^34^, the neutrons reaching the door belong to the low energy range. This highlights the need for a material capable of absorbing thermal neutrons. Also, it has been observed that compared to an open door, a 10 cm thick door does not affect the energy of neutrons, but it significantly reduces their intensity. This conclusion can be extended to thicker thicknesses of the door, as well.

**Figure 4:**
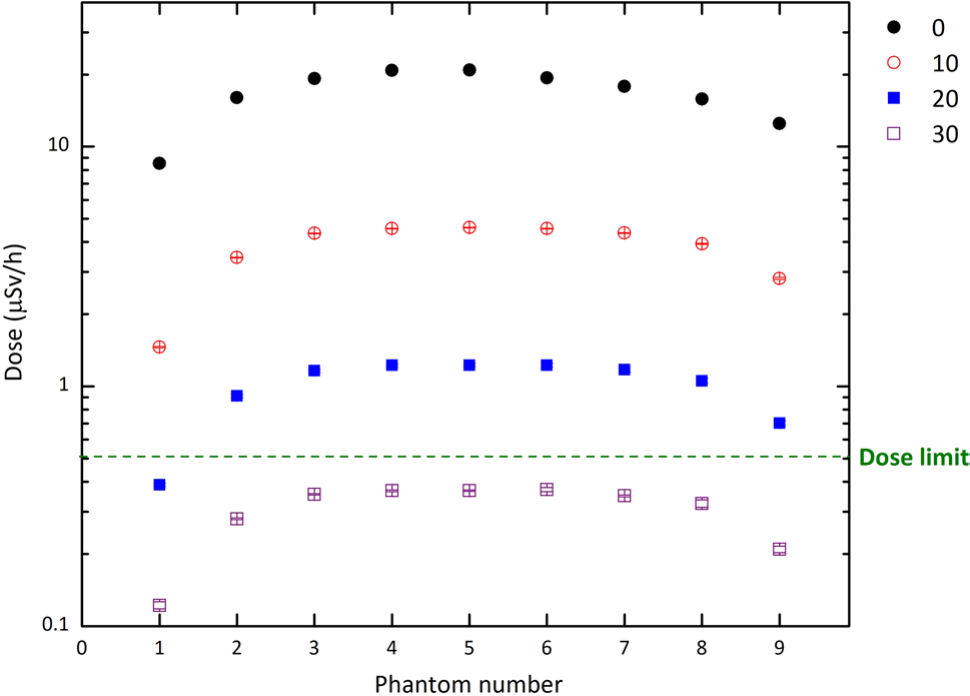
The neutron dose delivered to the phantoms for different thicknesses of the lead door. The thickness of 0 cm refers to the absence or being open of the door. Error bars show the relative uncertainties.

As mentioned earlier, the effectiveness of shielding materials was evaluated based on dose calculations in a phantom. To investigate the impact of lead, which is commonly used as a door material in treatment rooms, the dose delivered to the phantoms outside the room was calculated for different door thicknesses. The results were shown in Fig. 4. It is evident that compared to an open door, the dose rate in the central phantom outside the entrance door drops by a factor of 4.5 for a 10 cm thick lead. As the thickness of the lead door increases from 10 cm to 30 cm, the dose to the central phantom also decreases by a factor of 17 and 57, respectively. Also, the results indicate that a lead door with a thickness of approximately 30 cm can effectively decrease the dose to the outside phantoms to a level lower than the recommended limit. However, the use of such thick lead shielding is not ideal due to its heavy weight of about 23.4 tons caused by the door dimensions and the high density of lead. Furthermore, the high cost of this material makes the production of such a door expensive. To address this issue, a new shielding material was investigated, which consists of an optimized set of parameters listed in Table 1.

**Figure 5:**
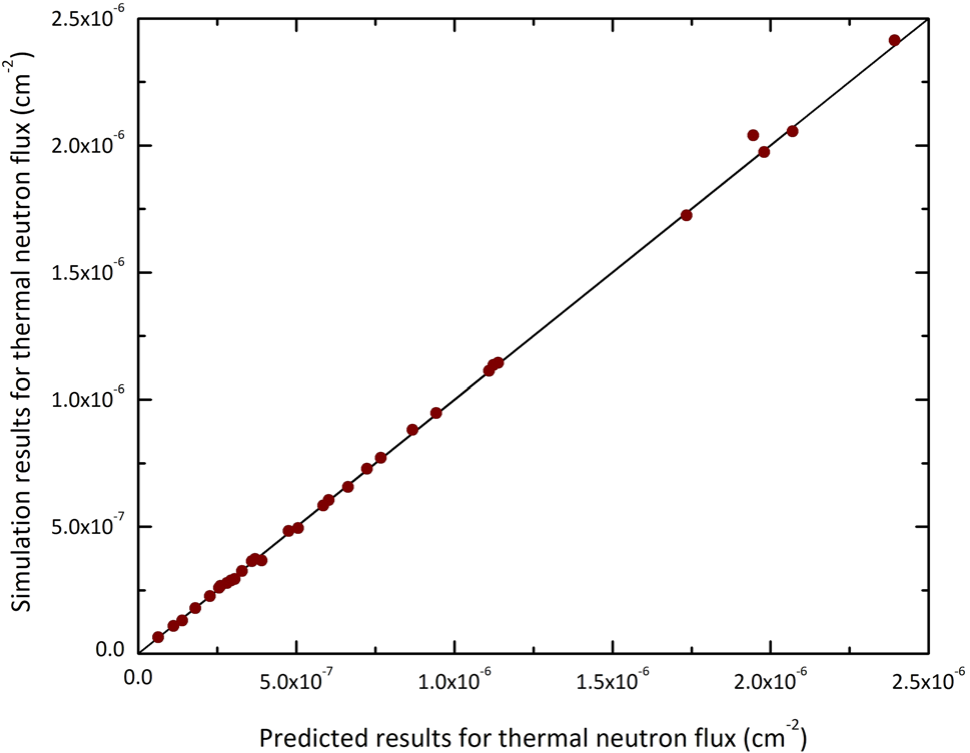
Comparison of the Monte Carlo and predicted results for training data corresponding to the thermal neutron flux. The values have been reported per neutron source particle.

First, to assess the efficacy of the material shielding, the thermal neutron flux after passing through the shields was calculated and the results were used as input for the ANN. The comparison between the Monte Carlo and predicted ANN results for testing data (Table 2) were shown in the regression diagram of Fig. 5. The degree to which the data points align with the line of equality ($x=y$) indicates the accuracy of the neural network predictions. It is apparent that the predicted values by the ANN agree well with those obtained from the simulations, demonstrating that an accurate estimation of the thermal neutron flux beyond the shield can be achieved. The thermal neutron flux is significant because it provides information not only about the intensity of the particles beyond the shield, but also serves as a measure related to the radiation dose outside the room.

**Figure 6:**
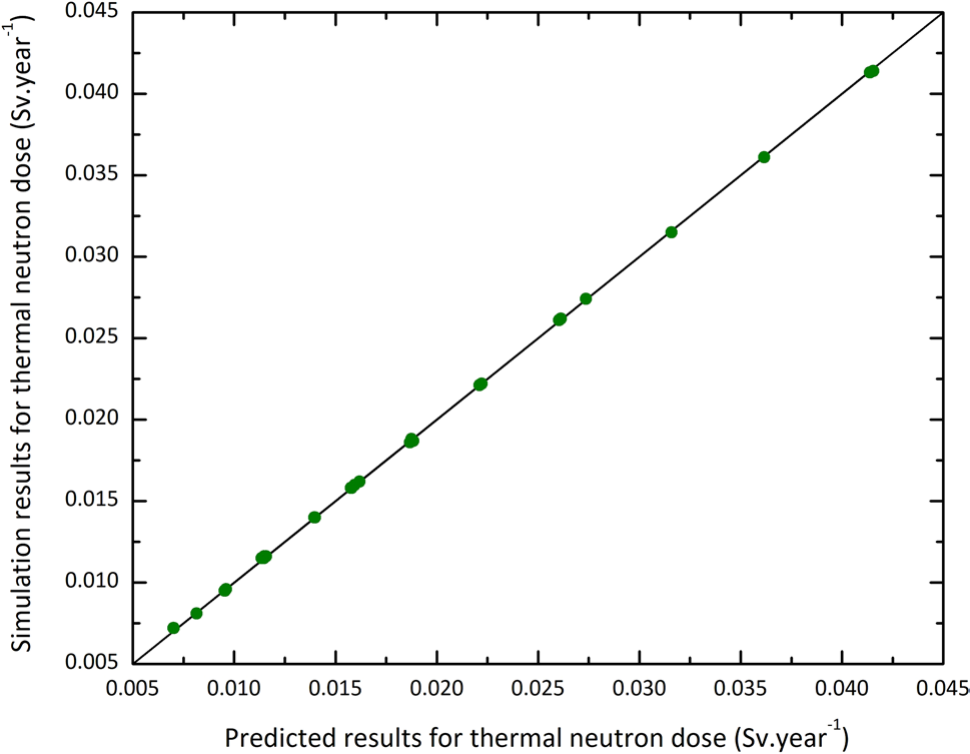
Comparison of the Monte Carlo and predicted results for training data corresponding to the thermal neutron dose after the shields.

**Figure 7:**
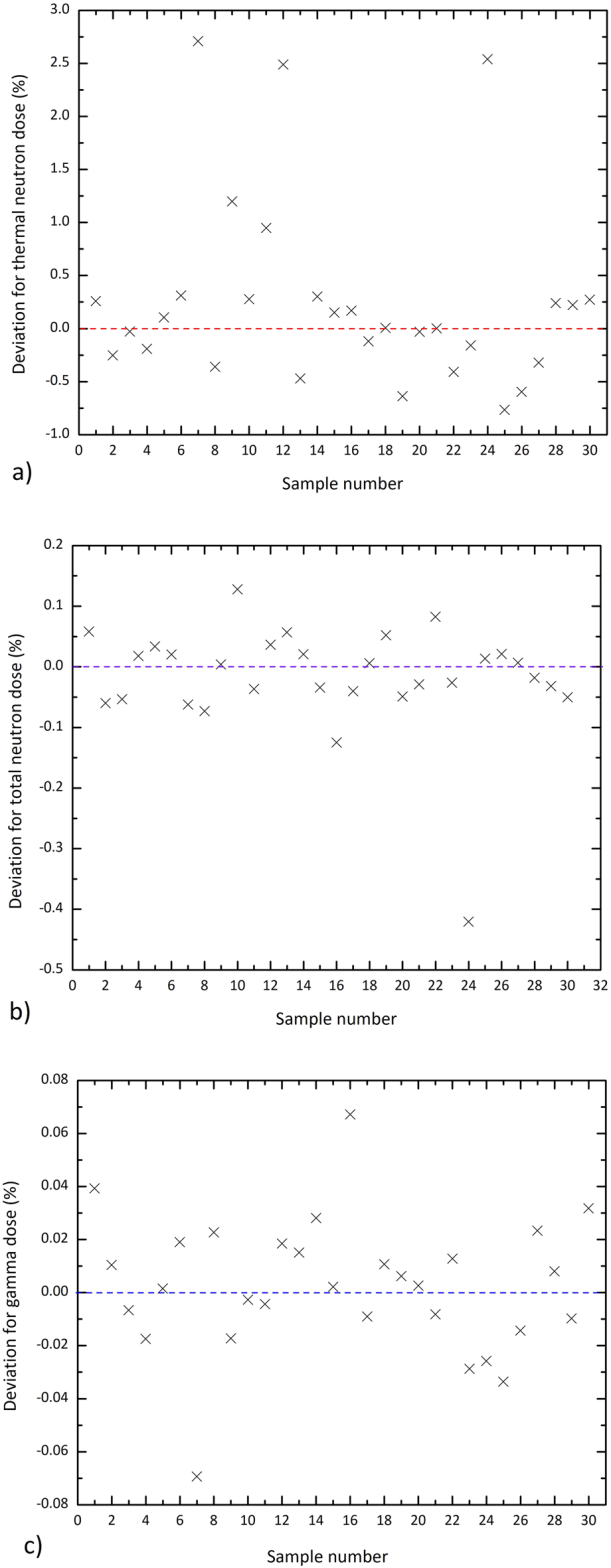
Deviation (in percent) between the ANN predicted values from those of the Monte Carlo code for (**a**) thermal neutron dose, (**b**) total neutron dose, and (**c**) gamma dose for the testing samples listed in Table 2.

Furthermore, calculations have been performed to determine the thermal neutron dose, total neutron dose, and gamma dose resulting from the neutrons passing through various sets of shields of Table 1. The results have been considered as the input data for training ANN. To evaluate the effectiveness of the ANN, a comparison has been made between the Monte Carlo simulation and predicted results for testing data. Figure 6 that shows the regression diagram for thermal neutron dose refers to the acceptable performance of the network. Similar calculations have been performed for the total neutron dose (i.e., neutron dose regardless of energy) and gamma dose. Figure 7 displays the deviation between the ANN predicted values from those of the Monte Carlo code for thermal neutron dose, total neutron dose, and gamma dose. As the results indicate, the absolute value of the deviations reaches the maximum values of about 2.7%, 0.015%, and 0.08% for thermal neutron dose, total neutron dose, and gamma dose, respectively.

**Figure 8:**
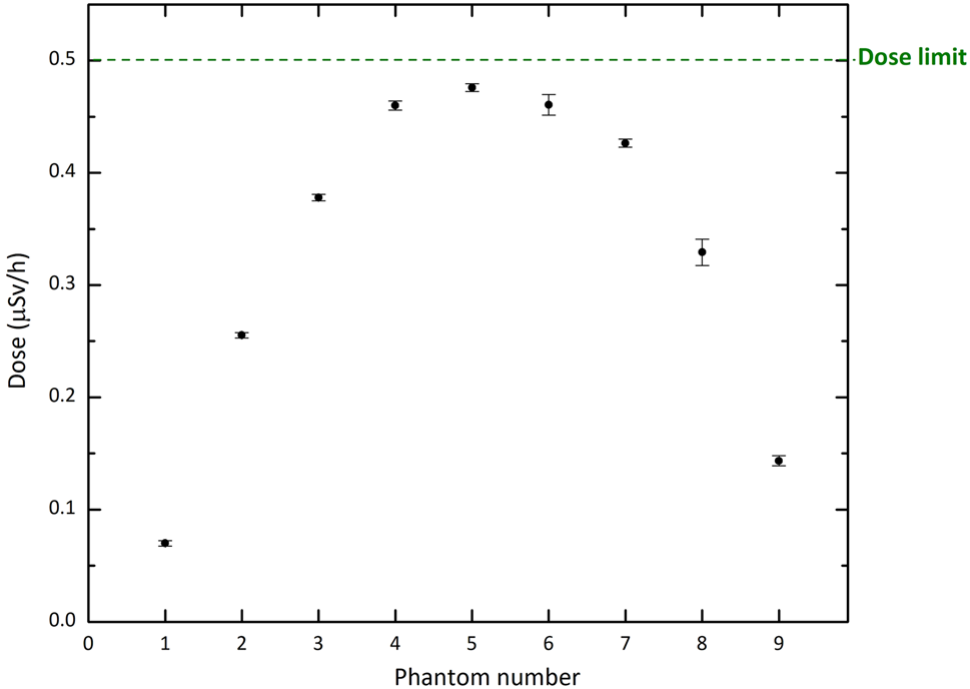
The neutron dose in the phantoms for the final shielding material suggested through ANN calculations. Error bars show the relative uncertainties.

**Figure 9:**
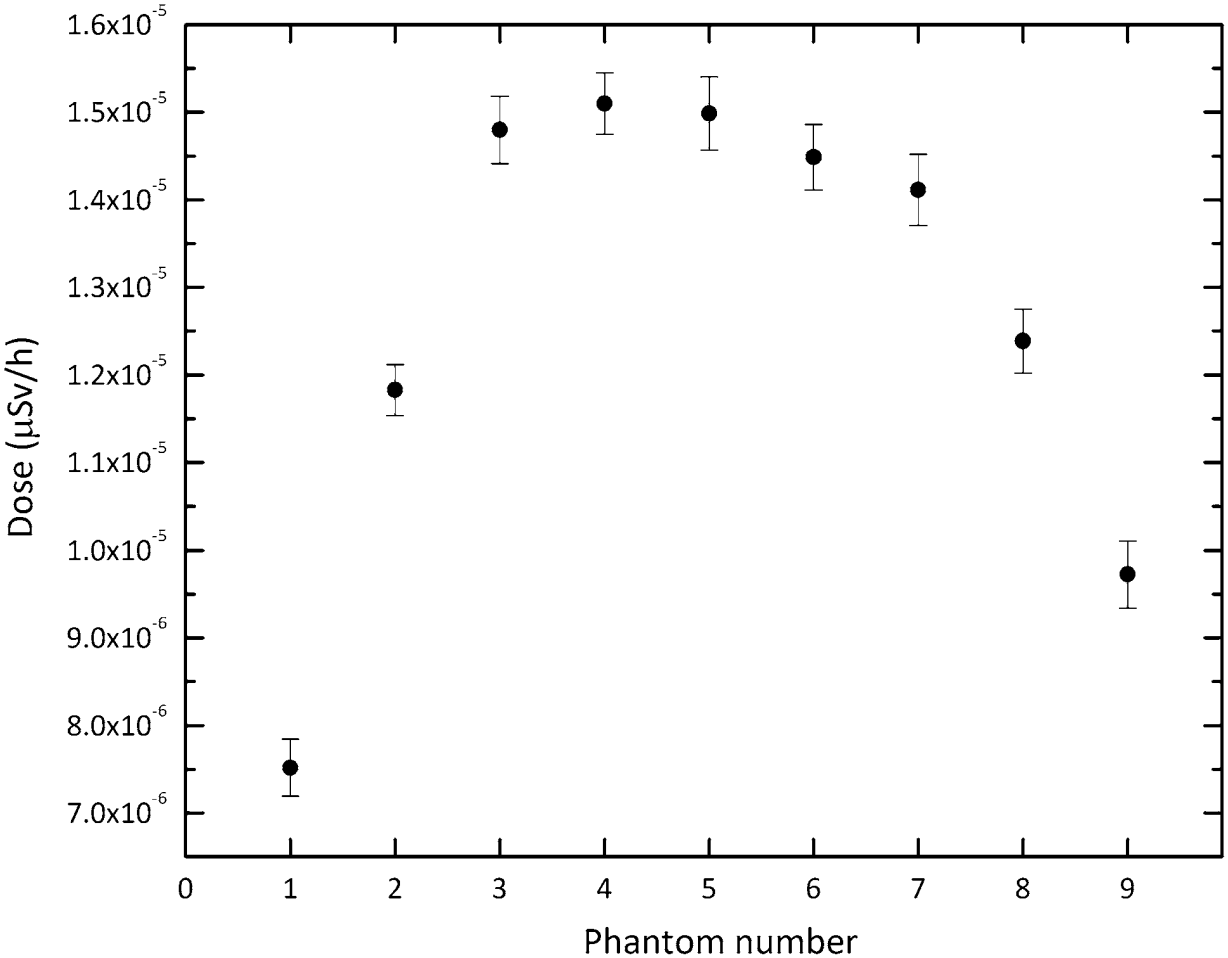
The gamma dose in the phantoms for the final shielding material suggested through ANN calculations. Error bars show the relative uncertainties.

Based on these results, our ANN can predict the dose values beyond the shield accurately for a given range of A to D parameters, eliminating the need for running the Monte carlo codes for all desired combinations of the parameters. After conducting multiple tests with various sets of the four parameters, a combination was identified that can effectively decrease the dose beyond the shield to the allowable level. The final material chosen for shielding thermal neutrons consists of the optimized values of 35% and 50% for A and B parameters, respectively. In the case of the C and D parameters, which represent the thickness of the layers, it is evident that as the values increase, the corresponding dose decreases. When considering the largest values for these parameters in our tested sets, namely 12 cm and 6 cm, the dose beyond the shield is approximately 0.22 $\upmu$Sv h$^{-1}$, which is well below the allowable limit of 0.5 $\upmu$Sv h$^{-1}$. A survey of dose values corresponding to the various sets of these parameters led to the proposal of using smaller thicknesses, specifically 10 cm and 4 cm, to achieve an appropriate shield in both dimensions and performance. To gain a preliminary understanding of how this material works as the entrance door of the BNCT treatment room, it has been simulated as the door material. Taking into account the neutron spectrum emitted from the BSA (as designed in the reference^34^), the dose values were computed for the phantoms located behind the door (see Fig. 1). The results are shown in Fig. 8. Based on the data, it is apparent that the dose values, even in the central phantom, are below the recommended limit. Also, Fig. 9 shows that the gamma dose values are considerably far from the dose limit. This is a promising outcome that suggests the proposed shield is effective in reducing occupational radiation exposure and can improve the safety of the BNCT treatment rooms.

Also, the dose values have been compared with those after passing the lead door reported in Fig. 4. Table 3 shows the deviation between these values and clarifies that the optimized shield can work more effectively than various lead thicknesses in reducing the dose values. Based on the results, it appears that the final shield suggested based on the ANN data outperforms lead as the entrance room door material. This is because it not only reduces the thermal neutron dose, but also weighs less due to its lower density, making it a suitable option for use in hospitals and BNCT radiation therapy rooms.

**Table 3:** Deviations (in percent) between the dose values delivered to the phantoms behind the door of the optimized shielding material from those behind various thicknesses of lead.

Phantom number	0 cm	10 cm	20 cm	30 cm
1	$-99.2$	$-95.2$	$-82.1$	$-42.7$
2	$-98.4$	$-92.6$	$-72.0$	$-9.0$
3	$-98.0$	$-91.5$	$-67.4$	$-6.6$
4	$-97.8$	$-89.9$	$-62.4$	$-25.2$
5	$-97.6$	$-89.8$	$-61.0$	$-29.6$
6	$-97.7$	$-89.9$	$-62.5$	$-23.9$
7	$-97.6$	$-90.2$	$-63.6$	$-21.9$
8	$-98.4$	$-93.6$	$-76.3$	$-22.9$
9	$-98.8$	$-94.9$	$-79.6$	$-31.3$

### Uncertainties

The development of neural networks’s precise, and estimation of the uncertainty of the generated values is a challenging work, and investigating and analyzing the sources and categories of uncertainty is of interest to many researchers^38–40^. Generally, the uncertainty may be due to the lack of data in the measurement process (in the case of experimental works), or in the calculations (in the case of analytical/simulation works). If the training data do not cover all the ranges of the chosen parameters perfectly, the uncertainty will increase there. Therefore, this type of uncertainty, known as epistemic uncertainty, expresses the model’s uncertainty and can be reduced by collecting more samples to the training dataset in an appropriate way in all ranges, as much as possible. The second type, known as the aleatoric uncertainty, is due to the underlying process that generated the dataset or errors in the measurement/calculation process. This uncertainty can not be reduced by observing/calculating more data.

**Figure 10:**
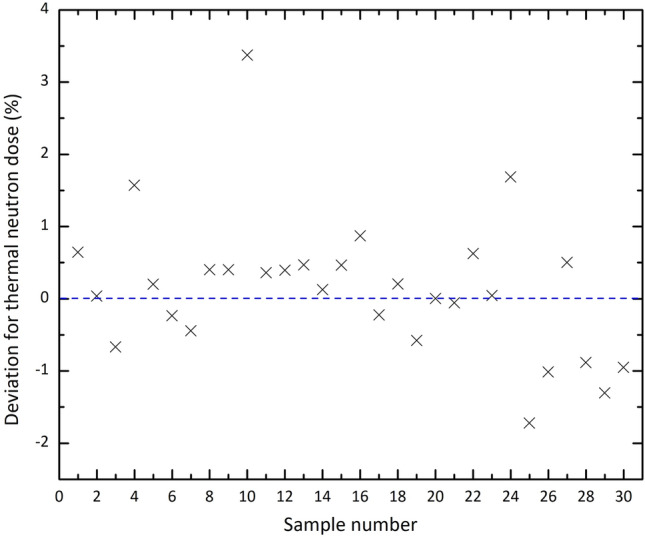
Deviation (in percent) between the ANN predicted values of thermal neutron dose from those of the Monte Carlo code for the testing samples listed in Table 2 in which a Gaussian distribution of random numbers with the typical width of 5 has been considered.

In order to check the stability of our ANN against the first type of uncertainty, the uncertainties have been applied to the testing data manually. For this purpose, a Gaussian distribution of random numbers with various widths has been applied to the testing data presented in Table 2. As an example of these investigations, Fig. 10 shows the deviation (in percent) between the ANN predicted thermal neutron dose from those of the simulations for the testing samples with the typical Gaussian distribution width of 5. As the results display, the deviations mainly range between about $-2$% to 2%, and the absolute value of the deviations reaches the maximum value of about 3.5%. The results are promising for using this ANN to predict unknown desired values and to get a useful outlook of the arrangement of the four parameters on the final results because of the ability to test the problem for various sets.

## Conclusions

In order to achieve the objective of the work, the study explored the development of an optimized thermal neutron shielding material to be used as an alternative to traditional lead material for the entrance door of BNCT treatment rooms. The study tested 720 different sets of four parameters, including the weight fractions of polyethylene and boric acid in the polymer composite, as well as the thickness of the polyethylene as the first layer and the thickness of the composite as the second layer, using the Geant4 Monte Carlo code. Though the results were encouraging, taking into account the disadvantages such as the time-consuming running of the Monte Carlo codes, using ANN for prediction of the dose/flux results of various sets was proposed. The collected data was used to train the neural network. To assess the accuracy of the ANN, a comparison was made between the Monte Carlo simulation and predicted results. It was found that the ANN predictions closely matched those of the simulations, with deviations of up to 2.7%. The data obtained from the trained neural network was utilized to achieve appropriate values for the four parameters involved in our thermal neutron shield.

Based on the recommendations of the International Commission on Radiological Protection and assuming a 40-hour work per week, the average dose limit for occupational workers was set at 0.5 $\upmu$Sv h$^{-1}$. The results show that when BNCT treatment proceeded with the previously designed neutron spectrum for deep tumors, the dose rate outside the entrance door of the treatment room, which was suggested using ANN, is well below the recommended dose limit. This finding is quite promising as it confirms that the 14 cm shield, consisting of a 10 cm thickness of polyethylene with a density of 0.9 g cm$^{-3}$ followed by a 4 cm thickness of polymer composite with a density of 1.185 g cm$^{-3}$ containing 35% polyethylene and 50% boric acid, performs even better than a 30 cm thickness of lead with a density of 11.34 g cm$^{-3}$, which weighs approximately 23.4 t.

Shielding neutron radiation is a challenging process due to its chargeless nature, which sets it apart from other types of radiation. Furthermore, using traditional optimization methods for predicting the behavior of thermal neutron shielding is difficult, time-consuming, and should be done in successive separate steps. A trained neural network model could easily and accurately predict the optimized shield. It can be concluded that ANN have great potential to be used as a flexible and effective solution in this context and can successfully be applied to find the optimal shield with consideration of multiple parameters.

## Data Availability

The datasets used and analysed during the current study available from the corresponding author on reasonable request.
